# Comparative Effects of BCI-Based Attention Training, Methylphenidate, and Citicoline on Attention and Executive Function in School-Age Children: A Quasi-Experimental Study

**DOI:** 10.3390/medicina62030448

**Published:** 2026-02-27

**Authors:** Serkan Turan, Remzi Oğulcan Çıray

**Affiliations:** 1 Child and Adolescent Psychiatry Unit, Bursa Uludag University, 16110 Bursa, Turkey; 2 Child and Adolescent Psychiatry Unit, Dokuz Eylül University, 35390 İzmir, Turkey; remziogulcanciray@gmail.com

**Keywords:** brain–computer interface, attention training, methylphenidate, citicoline, cognitive training, executive functions, CPT-3, children

## Abstract

*Background and Objectives*: Attention-Deficit Hyperactivity Disorder (ADHD) is a neurological condition characterized by cognitive task difficulty, impulsivity, hyperactivity and loss of attention. This study compared four approaches for improving attention and related skills in school-age children: COGO Brain–Computer Interface (BCI)-based attention training, methylphenidate, citicoline, and their combined use. *Materials and Methods*: A quasi-experimental pre–post design was used with four groups: COGO + methylphenidate (*n* = 44), COGO + citicoline (*n* = 44), COGO-only (*n* = 44), and citicoline-only (*n* = 42). Children completed baseline and post-treatment assessments, including the CPT-3 and several behavioral and emotional rating scales. Analyses included paired *t*-tests, ANCOVA, and repeated-measures ANOVA, adjusting for age. *Results*: The strongest improvements appeared in the COGO + methylphenidate group, especially in measures of sustained attention and reaction time consistency. The COGO + citicoline group showed clearer gains in inhibitory control (fewer commission errors) and reductions in anxiety/emotional symptoms. The COGO-only and citicoline-only groups showed little to no measurable change. Despite these within-group patterns, there were no significant differences between groups on CPT-3 outcomes or behavioral/emotional scales. *Conclusions*: This trial showed that combining COGO-based attention training with medication is both feasible and well-tolerated in children with attention and executive function difficulties. Moreover, the integrated approach produced measurable improvements across attentional performance and behavioral regulation domains.

## 1. Introduction

Attention-deficit/hyperactivity disorder (ADHD) is one of the most common neurodevelopmental disorders of childhood, characterized by age-inappropriate levels of inattention, hyperactivity, and impulsivity that substantially impair academic performance, behavioral regulation, and socio-emotional functioning [1]. The core attentional deficits associated with ADHD—including impaired sustained attention, increased reaction time variability, and deficits in inhibitory control—reflect disruptions in fronto-striatal and fronto-parietal networks that support cognitive control [2]. These neurocognitive vulnerabilities are not only central to the diagnosis of ADHD but also extend to many children referred for learning difficulties, academic underachievement, and emotional dysregulation, underscoring the need for effective interventions that can target both behavioral symptoms and underlying cognitive mechanisms.

Pharmacological treatment, particularly with stimulant medications such as methylphenidate, remains the first-line evidence-based intervention for ADHD and has been shown to produce robust short-term improvements in attention and executive functioning through dopaminergic and noradrenergic modulation [3]. Despite their established efficacy, stimulant medications present several limitations, including variable individual response, concerns about tolerability, adherence difficulties, and persistent hesitation among families regarding long-term reliance on pharmacotherapy [4,5]. Moreover, improvements in cognitive performance with stimulants may be task-dependent; for example, while reaction time variability on a simple Continuous Performance Task (CPT) X-task can be reduced by medication and relates to ADHD severity, more complex tasks such as the CPT A-X-task show less sensitivity to stimulant effects, highlighting that enhancements in sustained attention may be partial or context-specific [6]. These factors have intensified interest in complementary or alternative interventions that target ADHD-related cognitive deficits through different mechanisms.

The search for innovative treatments has led to renewed attention toward neurocognitive, neuromodulatory, and nutraceutical approaches [7]. Among nutraceuticals, citicoline (CDP-choline) has been explored due to its role in phospholipid metabolism, neuronal membrane repair, and modulation of dopaminergic pathways implicated in attention regulation [8]. However, evidence in pediatric populations remains preliminary. The most recent randomized pediatric trial found no significant advantage over placebo for core ADHD symptoms, although modest benefits were observed in select cognitive parameters [9]. Thus, citicoline is currently considered an experimental adjunctive agent rather than a stand-alone treatment.

In parallel, electroencephalography (EEG)-based neurofeedback (NFB) and Brain–Computer Interface (BCI)-assisted attention training have emerged as promising non-pharmacological approaches aimed at enhancing attentional control through operant modulation of neural activity. EEG-NFB trains individuals to modulate specific neurophysiological markers—such as theta/beta ratios, sensorimotor rhythms, or slow cortical potentials—by providing real-time feedback linked to EEG signals [10,11]. These protocols target neural substrates critical for sustained attention and inhibitory control, and early randomized controlled trials reported symptom reductions comparable in some cases to those achieved with stimulant medication [12]. More recent meta-analyses suggest small-to-moderate improvements in ADHD symptoms, though debate continues due to methodological variability, challenges in blinding, and differences in control conditions [12,13].

BCI-based attention training represents a newer generation of EEG-driven interventions that incorporate adaptive gamified tasks controlled by real-time neural signals [14]. Unlike traditional neurofeedback, BCI attention systems modify in-game events, task progression, or reinforcement contingencies directly based on fluctuations in EEG markers of attention. This design increases ecological validity and motivation in pediatric populations and allows delivery in accessible formats, including home-based programs [15]. Although early data support their feasibility and acceptability, evidence on cognitive outcomes remains limited, and direct comparisons with pharmacological or nutraceutical interventions are scarce.

Given these developments, integrating BCI-based attention training with pharmacological or nutraceutical agents represents a compelling next step in ADHD intervention research. The combination of stimulant medication—which enhances neurotransmitter availability—and BCI training—which targets neurofunctional self-regulation—may produce additive or synergistic effects on sustained attention and executive functioning [16,17]. Conversely, pairing BCI training with citicoline offers the potential for a non-stimulant multimodal option, though this has not been systematically evaluated. Despite theoretical rationale, empirical data comparing single-modality and combined interventions remain extremely limited. To date, few studies have directly compared BCI-based attention training with both stimulant and non-stimulant cognitive enhancers within the same clinical framework, making this investigation one of the first attempts to quantify the relative and combined benefits of these modalities.

Finally, the use of both behavioral rating scales and performance-based neuropsychological measures is crucial for accurately assessing treatment effects. Continuous Performance Tests such as the CPT-3 offer sensitive indices of omission errors, commission errors, reaction time variability, and vigilance, making them particularly well suited for evaluating attentional changes across interventions [18]. Integrating these measures with standardized parent-report scales offers a more comprehensive picture of treatment response.

The present study addresses critical gaps in the literature by comparing four intervention modalities—BCI-based attention training alone, BCI combined with methylphenidate, BCI combined with citicoline, and citicoline monotherapy—using a quasi-experimental pre–post design. By evaluating changes across CPT-3 performance metrics and multi-domain behavioral/emotional assessments (SNAP-IV, SCT, RCADS, SDQ, BRIEF), this study aims to clarify the relative and combined effects of novel and established treatments.

## 2. Materials and Methods

This research employed a quasi-experimental, non-randomized, prospective pre–post design. Participants were allocated into one of four parallel treatment conditions (COGO + Methylphenidate, COGO + Citicoline, COGO-only, Citicoline-only). Because treatment allocation followed clinical decision-making rather than random assignment, the study reflects a naturalistic clinical practice environment. All participants were assessed at baseline and at the end of the standardized intervention period. A priori sample-size calculations were not conducted; instead, group sizes reflected the number of eligible participants presenting during the study period. However, the final sample size (*n* = 174) provides adequate power for within-group analyses and medium effect detection in repeated-measures models. Clinical trial registration: https://www.clinicaltrials.gov; NCT07333339.

### 2.1. Participants

All cases consisted of school-aged children referred for attentional difficulties and cognitive-performance concerns. Group sample sizes ranged between 42 and 44. Sociodemographic variables—including age, gender distribution, and academic grade—were collected at baseline. A statistically significant difference was detected in age across groups (F = 3.3, *p* = 0.023). No significant differences emerged for gender or other baseline characteristics. Due to this imbalance, age was included as a covariate in all subsequent ANCOVA analyses.

### 2.2. Intervention Procedures

**Figure 1:**
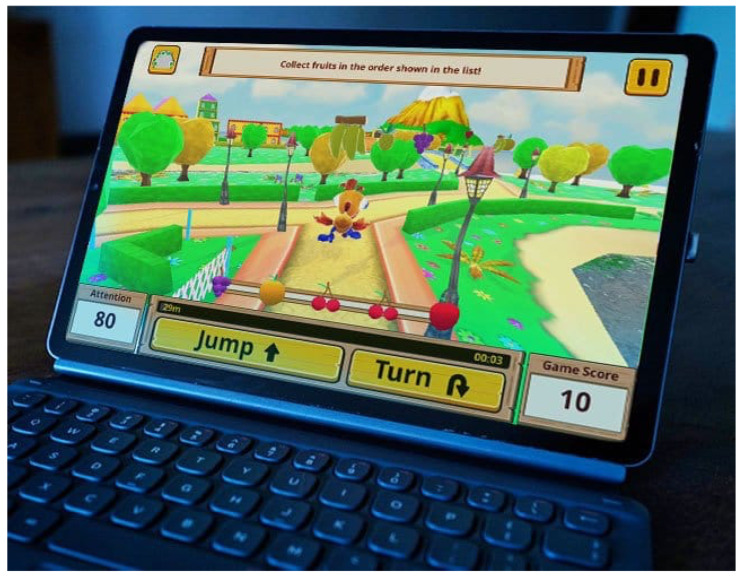
Overview of the Cogoland gameplay design across increasing task difficulty levels.

COGO Cognitive Training Program: The COGO Cognitive Training Program is typically delivered over an 8-week structured schedule, designed to provide consistent and progressive cognitive stimulation. Across this period, participants complete three training sessions per week (total of 24 sessions), each incorporating attention-focused tasks that adapt to the child’s performance in real time (Figure 1). Cogoland is a BCI-based attention training platform in which real-time EEG-derived attentional markers dynamically modulate gameplay difficulty and reinforcement contingencies. The system translates fluctuations in sustained attention and response engagement into adaptive in-game feedback, allowing repeated practice of attentional control within a structured, gamified environment. The program utilizes game-based exercises that translate neurocognitive processes—such as sustained attention, working memory, and inhibitory control—into engaging, feedback-driven activities. This systematic 8-week framework allows for incremental skill acquisition while maintaining motivation through short, repeatable sessions. Evidence from feasibility studies indicates that children can follow this schedule with minimal support, and that regular participation across the 8-week protocol is associated with measurable improvements in attentional regulation and related behavioral domains.

Cogoland features a three-tiered difficulty structure designed to progressively challenge players’ cognitive and motor skills. At this stage, players use designated keyboard keys—such as the jump command—to help the avatar reach the target items. Each training session consists of two 10 min gameplay segments separated by a brief rest interval.

### 2.3. Pharmacological Interventions

Participants assigned to medication groups received one of the following agents:

- Methylphenidate (Mph): Administered in clinically appropriate doses based on pediatric guidelines and clinical judgment.
- Citicoline: Delivered in standardized age-appropriate dosing as an adjunctive neurocognitive enhancer.

### 2.4. Other Medications and Confounding Factors

Families were asked to report all medications used by the child during the study. Children receiving stable doses of medications unrelated to attentional functioning (e.g., antihistamines, asthma medications) were allowed to participate; however, no participants were using additional psychotropic agents. Potential confounders—including sleep difficulties, comorbid emotional symptoms, and grade level—were documented, and analyses were adjusted for age.

### 2.5. Outcome Measures

The primary outcome measure of the study was attentional performance, assessed using the Conners Continuous Performance Test–Third Edition (CPT-3). This computerized test provided T-score-based indices of multiple attention domains, including omission and commission errors, perseverative responses, hit reaction time (HRT), the standard deviation of HRT, overall response variability, and changes in HRT across task blocks and across different inter-stimulus intervals. Together, these metrics offered a comprehensive profile of sustained attention, inhibitory control, and response consistency. Secondary outcomes were evaluated using a broad set of standardized behavioral and emotional measures. These included the SNAP-IV scales for inattention and hyperactivity, the Sluggish Cognitive Tempo (SCT) scale, and the Revised Child Anxiety and Depression Scale (RCADS) to assess internalizing symptoms. Additionally, general behavioral functioning was captured through the Strengths and Difficulties Questionnaire (SDQ), while executive functioning was examined using the Behavior Rating Inventory of Executive Function (BRIEF), including its Behavioral Regulation Index, Metacognition Index, and Global Executive Composite scores. All measures were administered both before and after the intervention to evaluate changes attributable to the training program.

### 2.6. Statistical Analysis

Data were analyzed using SPSS 26.0. Paired-samples *t*-tests were conducted within each intervention group to examine pre–post differences in CPT-3 performance. Repeated-measures ANOVA was used to assess group-by-time interactions in SNAP-IV, SCT, RCADS, SDQ, and BRIEF. Between-group comparisons for CPT-3 variables were made using one-way ANOVA. Effect sizes were reported using Cohen’s d for paired *t*-tests and partial η^2^ for ANOVAs. Statistical significance was set at *p* < 0.05.

## 3. Results

### 3.1. Participant Characteristics

Four intervention groups were included in the analyses: COGO + Methylphenidate (Mph), COGO + Citicoline, COGO-only, and Citicoline-only. Group sizes ranged from 42 to 44 participants. A significant difference emerged in age across groups (F(3145) = 3.30, *p* = 0.023), whereas gender distribution and grade level did not differ significantly (all *p* > 0.05) (Table 1). Baseline clinical scale scores were comparable across groups, except for prosocial behavior (*p* = 0.005). Due to age differences, all subsequent ANCOVA analyses were adjusted for age.

### 3.2. Primary Outcomes

Across the four intervention conditions, patterns of change in CPT-3 attention-performance indices demonstrated notable variability, with the most prominent gains emerging in the combined intervention groups. Overall, methylphenidate-augmented COGO training produced the strongest improvements across multiple domains of sustained attention and response consistency, whereas citicoline-augmented COGO training showed selective but meaningful benefits. In contrast, single-modality interventions—COGO alone and citicoline alone—yielded minimal or no measurable changes (Table 2).

Participants receiving the combined COGO + methylphenidate intervention showed the most robust and consistent improvements. Significant reductions in omission errors (t = 2.30, *p* = 0.032, d = 0.49) indicated better sustained attention and reduced lapses in vigilance. Additionally, a significant improvement in HRT ISI change (t = 3.24, *p* = 0.004, d = 0.69) reflected enhanced stability of reaction time across different inter-stimulus intervals, suggesting greater attentional adaptability during task demands. Although not statistically significant, moderate improvements were also seen in commission errors, HRT, HRT SD, and variability (effect sizes ranging from d = 0.34 to 0.54). Together, these findings suggest that the addition of methylphenidate enhanced the cognitive effects of BCI-based attention training, particularly in domains reflecting vigilant attention and temporal response consistency (see Table 2).

The COGO + citicoline group demonstrated a distinct improvement pattern. This combination led to a significant reduction in commission errors (t = 2.59, *p* = 0.017, d = 0.55), indicating improved inhibitory control and reduced impulsive errors. A trend toward reduced perseveration errors (t = 1.91, *p* = 0.070, d = 0.41) suggested potential gains in cognitive flexibility and response monitoring. The magnitude of change across other indices was smaller (d = 0.11–0.41), indicating that the benefits of citicoline, when combined with COGO, were more specific to inhibition-related attentional processes rather than broad improvements across the attention spectrum.

The only COGO training condition produced limited changes. No CPT-3 variable reached statistical significance (all *p* > 0.05), and effect sizes remained small across omission, commission, reaction time, and variability measures (d < 0.42). These findings suggest that, in this naturalistic clinical context, COGO training without adjunctive pharmacological or nutraceutical support may have insufficient intensity or duration to produce measurable improvements in computerized attention performance.

Citicoline monotherapy generated minimal changes in CPT-3 performance. Small but non-significant improvements were observed in omission and perseveration errors (d ≈ 0.33), while all other indices—including commission errors, HRT, variability, and block/ISI change variables—remained largely stable. Overall, citicoline alone did not appear to substantially enhance attentional performance as measured by CPT-3 in this sample (see Table 2).

Despite the within-group improvements observed particularly in the combined intervention arms, one-way ANOVA revealed no significant between-group differences across any CPT-3 outcome (all *p* > 0.15, partial η^2^ = 0.01–0.06). The absence of group-level differences suggests that while certain interventions may promote meaningful individual improvements, variability within groups and the quasi-experimental design may limit the detectability of treatment-specific effects at the between-group level (Table 3).

### 3.3. Secondary Outcomes

**Figure 2:**
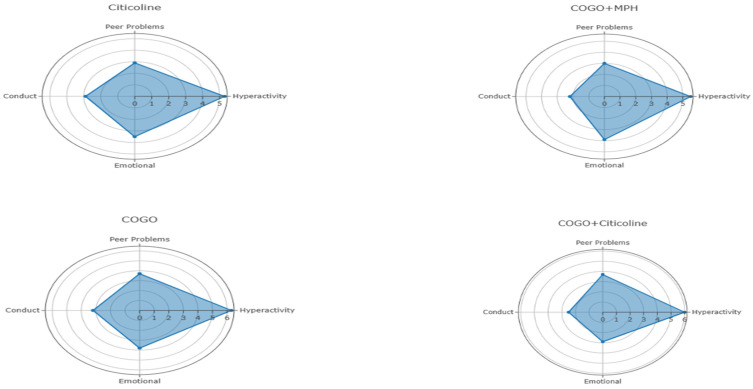
Comparison of participants in terms of behavioral problems after treatment.

Secondary behavioral, emotional, and executive function outcomes across the four intervention groups are summarized in Table 4, while the corresponding repeated-measures ANOVA results examining group-by-time interactions are presented in Table 4. A graphical depiction of post-treatment behavioral profiles is provided in Figure 2. Figure 2 presents a comparative visualization of post-treatment behavioral problem scores across the four intervention groups. Lower scores indicate fewer behavioral difficulties, allowing direct comparison of overall behavioral profiles following each treatment condition. This figure is intended to complement the tabulated results by providing an integrated visual summary of group-level behavioral outcomes.

Across the full sample, pre–post changes on parent-reported measures were generally modest, with improvements occurring selectively within specific intervention groups rather than uniformly across all measures.

The COGO + methylphenidate group demonstrated the most consistent secondary-outcome improvements, aligning with the robust gains in CPT-3 performance. As shown in Table S1, significant reductions were observed in SDQ hyperactivity scores (*p* = 0.030), suggesting observable behavioral improvement beyond laboratory-based measures. Notably, several executive function domains measured by the BRIEF improved significantly, including inhibition (*p* = 0.032), planning/organization (*p* = 0.014), monitoring (*p* = 0.021), the Metacognition Index (*p* = 0.031), and the Global Executive Composite (*p* = 0.036). These findings indicate that methylphenidate may enhance the functional transfer of BCI-based cognitive gains to real-world executive functioning. To complement statistical analyses, clinically meaningful change was examined using predefined criteria. A clinically relevant response was defined as an improvement of ≥1 standard deviation from baseline on BRIEF indices or a shift toward subclinical ranges. A marked improvement was observed in the COGO + methylphenidate group, particularly with respect to the Global Executive Composite and Metacognition Index scores, compared with the other groups (see Table S1). Although post-intervention BRIEF scores frequently remained within the clinically elevated range, the observed reductions reflect substantial functional gains rather than full symptom remission, consistent with real-world treatment outcomes in pediatric ADHD. Exploratory analyses were conducted to examine whether participants demonstrating the largest improvements were evenly distributed across intervention conditions. It was demonstrated that the COGO + methylphenidate group exhibited significantly better performance on the CPT-3 (particularly with respect to Omissions and HRT ISI change) and on the BRIEF subscales (especially the Emotional subscale). This pattern was considered likely to have contributed to the observed improvement in treatment response.

Exploratory analyses indicated that higher baseline severity was associated with greater magnitude of improvement on select attentional and executive function measures. According to the exploratory analysis, 14 participants (16%) with the lowest baseline Omissions scores demonstrated performance improvements ranging from 33% to 65%. Similarly, 8 participants (9.1%) with the poorest baseline HRT ISI Change scores exhibited improvements between 32% and 52%, of whom 6 were in the COGO + methylphenidate group and 2 were in the COGO + citicoline. For Commission errors, the 4 participants (4.4%) with the lowest baseline performance (within the COGO + citicoline group) showed improvements ranging between 16% and 33%. A responder analysis was conducted to identify participants demonstrating clinically meaningful improvement on key CPT-3 indices. Responders were defined as individuals showing a ≥30% improvement from baseline on at least one primary attentional outcome (Omissions, HRT ISI Change, or Commissions), a threshold commonly used to reflect clinically relevant change beyond measurement variability. This pattern was most evident in the combined intervention groups, suggesting the presence of a subgroup of “super-responders” characterized by more pronounced initial impairment. Despite these improvements, SNAP-IV inattention and hyperactivity scores did not change significantly, consistent with the limited group-by-time effects reported in Table 4. Nevertheless, the pattern suggests that combined COGO + methylphenidate may yield meaningful gains in higher-order executive operations that parents can observe in daily behavior.

Children in the COGO + citicoline group exhibited a different but clinically relevant pattern of improvement. As detailed in Table S1, significant reductions were found in RCADS total scores (*p* = 0.016), indicating decreased anxiety/depressive symptoms. Similarly, SDQ emotional problems decreased significantly (*p* = 0.026), suggesting improved emotional regulation and reduced internalizing symptoms. On BRIEF, the emotional control subscale improved significantly (*p* = 0.012), underscoring potential benefits of citicoline augmentation for affective regulation and behavioral flexibility. These improvements occurred despite the lack of significant change in attentional indices beyond commission errors, implying that citicoline’s impact may be more prominent in emotional/affective domains.

Only COGO group demonstrated minimal improvements across behavioral and emotional scales. As shown in Table S1, only a trend-level reduction was observed for SDQ emotional symptoms (*p* = 0.054), while all other SDQ, SNAP-IV, and RCADS scores remained unchanged. No BRIEF indices showed significant improvement. This pattern parallels the CPT-3 findings, suggesting that without adjunctive agents, the COGO program may produce only limited behavioral or executive function changes in a naturalistic clinical setting.

The citicoline-only group demonstrated stable scores across nearly all scales in Table S1, with no significant pre–post improvements. Small, non-significant changes appeared in SDQ hyperactivity and BRIEF emotional control, but these did not approach statistical significance. Overall, citicoline monotherapy did not yield measurable changes in attentional, behavioral, or emotional dimensions within the study period.

Repeated-measures ANOVA results presented in Table 4 indicate that no significant group-by-time interactions emerged for any behavioral, emotional, or executive function measure (all *p* > 0.13; partial η^2^ ≤ 0.07). This suggests that while within-group improvements were present—particularly in COGO + methylphenidate and COGO + citicoline—the magnitude of these changes did not differ significantly when groups were compared directly.

### 3.4. Graphical Representation

Figure 2 provides a visual comparison of post-treatment behavioral problem scores across the four groups. The figure illustrates a slight reduction in behavioral difficulties for the combined intervention groups compared to single-modality interventions, consistent with the selective improvements reported in Table 4, though these differences did not achieve statistical significance at the group-by-time level.

## 4. Discussion

The present study examined the comparative effects of COGO-based BCI attention training, methylphenidate, and citicoline—both as monotherapies and in combination—on attentional performance, executive functioning, and emotional outcomes in school-aged children exhibiting attentional difficulties. The overall pattern of findings suggests that multimodal treatment approaches may yield the most meaningful improvements, although variability across outcome domains underscores the complex and multidimensional nature of attentional regulation in childhood. These results align with more recent conceptualizations of ADHD and attention-related problems, which emphasize heterogeneous neurodevelopmental trajectories and multimodal therapeutic responsiveness [19,20].

The most pronounced improvements emerged in the COGO + methylphenidate group, which demonstrated significant gains in sustained attention and temporal response stability. Improvements in omission errors and HRT consistency are in line with contemporary neurobiological models showing that methylphenidate enhances activity in cortico-striatal loops responsible for attentional persistence, neuromodulatory gain, and cognitive stability [21]. Several neuroimaging studies have documented that stimulant medications normalize hypoactivation patterns in the dorsal attention network and improve the efficiency of prefrontal cortical signaling [22]. Considering that COGO training adaptively responds to real-time EEG markers of attention, stimulant-induced increases in neural signal fidelity may enhance the extent to which children can sustain engagement with the training protocol. This interaction between neurochemical optimization and neurofeedback-guided adaptive training may partially explain why the combined intervention produced improvements exceeding those of single-modality treatments.

The COGO + citicoline group exhibited a markedly different pattern, with improvements centered around inhibitory control and emotional regulation rather than sustained attention. Reductions in commission errors and emotional symptoms suggest specific benefits in regulating impulsivity and affect, consistent with recent evidence indicating that citicoline influences cholinergic and dopaminergic pathways implicated in cognitive inhibition and emotional modulation [23]. Experimental data further suggest that citicoline enhances phospholipid synthesis and neuronal membrane stability, potentially improving the efficiency of frontal networks involved in inhibitory control [24]. The emotional improvements observed in this group resonate with emerging findings showing that citicoline supplementation may impact affective systems through improved fronto-limbic connectivity and modulation of acetylcholine-mediated emotional regulation circuits [25]. Notably, these emotional benefits were not as evident in the methylphenidate group, which may suggest differential neuropharmacological targeting across combined modalities.

In contrast to these combined interventions, neither COGO alone nor citicoline alone produced substantial improvements. The limited effect of COGO-only training may reflect several contemporary concerns regarding BCI-based protocols. Recent meta-analyses have reported substantial heterogeneity in treatment effects across neurofeedback modalities, with improvements often contingent on training duration, fidelity, and patient-specific neurocognitive profiles [26]. Importantly, the training dose provided in the present study may have been insufficient when compared to higher-intensity neurofeedback and BCI interventions used in controlled trials [27]. Similarly, citicoline monotherapy produced minimal cognitive or behavioral change, consistent with recent findings showing that citicoline’s effects may be too subtle to yield clinically meaningful benefits without concurrent cognitive or behavioral engagement [28].

The lack of significant between-group differences across broader behavioral and executive function measures highlights the challenges of detecting treatment effects through parent-report instruments. Importantly, while executive function scores often remained above clinical cutoffs following intervention, the magnitude of improvement observed—particularly reductions exceeding one standard deviation—suggests meaningful functional change rather than simple statistical fluctuation. Recent research has underscored that performance-based tasks and neurophysiological indices often capture treatment-associated changes more sensitively than questionnaire-based ratings, particularly over short time periods [29]. Moreover, attentional difficulties in naturalistic pediatric samples are often accompanied by comorbidities such as anxiety, irritability, and learning challenges, which can dilute measurable group-level differences in behavior [30]. These factors may partially explain why CPT-derived markers showed clearer improvements than caregiver-reported scales. These exploratory findings should be interpreted as hypothesis-generating, but they raise the possibility that combined interventions may preferentially benefit a subset of children with specific neurocognitive profiles rather than exerting uniform effects across all participants.

Despite these methodological constraints, the findings bear important clinical implications. First, they support the growing argument that multimodal approaches—combining neurobiologically grounded pharmacotherapy with neuroplasticity-oriented training—may provide superior outcomes to single-modality interventions. Second, the differentiated improvement patterns across combination treatments highlight the potential for personalized intervention planning: children exhibiting predominantly attentional and executive deficits may benefit more from stimulant-enhanced BCI training, whereas children with prominent emotional dysregulation or inhibitory control problems may respond more favorably to citicoline-augmented training. This aligns with recent precision-psychiatry frameworks emphasizing individualized neurocognitive profiles to guide treatment selection [31]. Overall, the distinct improvement patterns across the combined treatment groups indicate that the selected adjunct treatment may differentially influence which cognitive or emotional functions benefit most from training. The pronounced improvements observed in the COGO + methylphenidate group should also be interpreted in light of developmental factors. This group was, on average, younger than the others, and younger children may exhibit greater neuroplastic responsiveness to combined pharmacological and cognitive interventions. Measures such as omission errors and HRT ISI Change are particularly sensitive to vigilance and adaptability, which may be more malleable earlier in development. Although age was statistically controlled, these findings highlight the need for future age-stratified or developmentally informed analyses. This underscores the importance of designing multimodal interventions that align with each child’s specific clinical needs, in line with contemporary precision-based approaches in pediatric neuropsychiatry.

Nevertheless, several limitations warrant consideration. The quasi-experimental allocation introduces potential selection biases, and although age was statistically controlled, residual confounding may remain. The intervention duration was relatively short, and emerging evidence suggests that neurofeedback and BCI interventions often require extended protocols (≥10–12 weeks) to reach their full therapeutic potential [13]. Additionally, the absence of objective adherence monitoring limits the interpretation of training fidelity. Future randomized, controlled, and longitudinal studies incorporating neurophysiological markers, real-time adherence tracking, and neuroimaging assessments would be essential to disentangle how different components of multimodal interventions contribute to cognitive and emotional outcomes. The absence of significant between-group differences should be interpreted with caution, as quasi-experimental allocation and variability in baseline age may have reduced statistical power to detect treatment-specific effects, even when meaningful within-group changes were present. The limited improvements observed in the COGO groups may reflect challenges in implementing the program among children with significant attention deficits and hyperactivity, who often struggle to maintain the level of focus required for optimal engagement. Although multiple attention-related scale scores improved in the MPH group, such gains were minimal with combined COGO or COGO alone, suggesting that difficulties in sustaining participation may have constrained its effectiveness. From a clinical perspective, these findings suggest that children with more severe baseline deficits may derive disproportionate benefit from multimodal interventions. However, given the exploratory nature of these analyses and the potential influence of regression to the mean, these observations should be interpreted cautiously and warrant confirmation in randomized, adequately powered trials. These implementation challenges should be carefully considered in future studies.

## 5. Conclusions

In conclusion, the findings suggest that combining COGO-based attention training with methylphenidate yields the most robust improvements in attentional performance, whereas combining citicoline with COGO appears particularly beneficial for emotional and inhibitory control outcomes. Single-modality interventions produced limited changes, underscoring the value of multimodal strategies. Future research may benefit from incorporating neurophysiological biomarkers (e.g., EEG spectral dynamics or connectivity indices) both as predictors of training responsiveness and as mechanistic indicators of how pharmacological agents interact with BCI-driven cognitive engagement. Although between-group differences were not statistically significant, within-group improvement patterns highlight clinically meaningful trends, warranting further investigation. These results support emerging perspectives advocating for integrated, neurobiologically informed, and personalized intervention models for attentional difficulties in childhood.

## Figures and Tables

**Table 1:** Sociodemographical and clinical characteristics of the study groups.

Variables	COGO + Mph	n	COGO + Citicoline	n	COGO	n	Citicoline	n	X^2^/F	*p*
Age	9.7 ± 2.4	44	10.4 ± 2.5	44	11.3 ± 2.8	44	11.3 ± 3.1	42	3.3	0.023 ^a^
Gender, n (%)		44		44		44		42	0.2	0.971
Female	15 (34.1)		14 (31.8)		16 (36.4)		15 (35.7)			
Male	29 (65.9)		30 (68.2)		28 (63.6)		27 (64.3)			
Grade	4.4 ± 2.6	38	4.8 ± 2.7	39	6.1 ± 3.1	40	6.2 ± 3.3	33	3.5	0.018 ^a^
RCADS ^b^	30.8 ± 19.6	38	24.4 ± 12.9	39	32.7 ± 16.8	40	32.2 ± 17.4	33	2.4	0.075
SNAP-IV ^b^										
Inattention	14.8 ± 5.3	38	13.6 ± 5.2	39	14.7 ± 4.6	40	13.3 ± 5.9	33	0.8	0.485
Hyperactivity	12.4 ± 5.1	38	12.3 ± 6.2	39	12.5 ± 6.0	40	11.8 ± 6.4	33	0.2	0.914
SCT Scale ^b^	21.4 ± 6.7	38	18.5 ± 5.4	39	22.1 ± 8.3	40	19.7 ± 6.4	33	2.3	0.076
SDQ ^b^										
Emotional	3.7 ± 2.9	38	3.2 ± 2.3	39	4.1 ± 2.6	40	3.9 ± 2.4	33	1.2	0.311
Conduct	2.4 ± 1.8	38	3.1 ± 2.0	39	3.4 ± 2.1	40	2.3 ± 1.5	33	2.8	**0.043**
Hyperactivity	6.2 ± 2.0	38	5.7 ± 2.5	39	6.0 ± 2.0	40	6.0 ± 2.4	33	0.3	0.849
Peer Problems	3.8 ± 2.2	38	2.8 ± 1.9	39	3.3 ± 2.1	40	3.2 ± 2.2	33	1.1	0.346
Prosocial Behavior	8.3 ± 1.6 ^a^	38	7.2 ± 1.9 ^b^	39	7.1 ± 1.8 ^b^	40	7.2 ± 2.1^b^	33	4.4	**0.005**
Total Difficulty	16.1 ± 6.3	38	14.8 ± 5.7	39	16.8 ± 6.0	40	15.3 ± 5.7	33	0.8	0.475
BRIEF ^b^										
Inhibition	28.8 ± 5.6	38	28.7 ± 7.4	39	29.6 ± 6.8	40	29.4 ± 6.3	33	0.1	0.960
Shifting	21.9 ± 4.1	38	22.0 ± 4.7	39	22.7 ± 4.2	40	22.8 ± 5.0	33	0.7	0.551
Emotional	20.1 ± 4.9	38	20.0 ± 5.2	39	21.8 ± 5.1	40	19.6 ± 4.6	33	1.4	0.244
Initiation	17.1 ± 3.9	38	16.9 ± 3.6	39	17.8 ± 2.8	40	16.6 ± 3.4	33	0.7	0.526
Working Memory	23.7 ± 4.6	38	23.3 ± 4.6	39	24.1 ± 4.2	40	23.6 ± 4.8	33	0.2	0.908
Planning	33.1 ± 1.3	38	32.0 ± 6.6	39	33.4 ± 6.2	40	32.2 ± 7.1	33	0.4	0.749
Organization	16.7 ± 4.8	38	17.3 ± 4.6	39	18.1 ± 3.9	40	16.4 ± 4.3	33	1.0	0.414
Monitoring	18.4 ± 3.3	38	17.5 ± 3.4	39	18.7 ± 2.8	40	18.0 ± 3.9	33	0.9	0.463
Behavioral Regulation	70.8 ± 12.5	38	70.8 ± 17.4	39	74.1 ± 13.6	40	71.8 ± 15.9	33	0.5	0.661
Metacognition Index	109.2 ±20.3	38	106.9± 20.2	39	112.0 ± 16.2	40	106.7 ± 20.6	33	0.6	0.626
Global Executive	180.0 ± 28.9	38	177.7 ± 32.6	39	186.1 ± 25.3	40	178.5 ± 34.2	33	0.6	0.643

^a^ The initial significance did not persist following post hoc tests. ^b^ ANCOVA, Adjusted for age and grade. BRIEF: Behavior Rating Inventory of Executive Function, RCADS: The Revised Child Anxiety and Depression Scale, SCT: sluggish cognitive tempo, SDQ: Strenghts and Difficulties Questionnaire, SNAP-IV: The Swanson, Nolan, and Pelham Rating Scale Different superscripts show post hoc differences. Bold values indicate statistical significance at 0.05 level.

**Table 2:** Within-group pre–post changes on CPT-3 T-scores across the four intervention conditions.

Intervention	CPT-3 (T Score)	Mean ± SD	Std. Error Mean	t	*p*	Cohen’s d
COGO + Mph						
	Omissions	9.91 ± 20.22	4.31	2.3	**0.032**	0.49
	Commissions	3.86 ± 10.4	2.22	1.74	0.096	0.37
	Perseverations	1.68 ± 16.8	3.58	0.47	0.641	0.1
	HRT	4.68 ± 8.6	1.83	2.55	0.643	0.54
	HRT SD	6.85 ± 20.5	4.37	1.59	0.126	0.34
	Variability	0.91 ± 18.1	3.85	0.24	0.816	0.05
	HRT ISI Change	11.41 ± 16.5	3.52	3.24	**0.004**	0.69
COGO + Citicoline						
	Omissions	4.86 ± 17.5	3.72	1.31	0.205	0.28
	Commissions	3.68 ± 6.7	1.42	2.59	**0.017**	0.55
	Perseverations	5.59 ± 13.4	2.93	1.91	0.07	0.41
	HRT	−2.41 ± 7.3	1.56	−1.54	0.138	−0.33
	HRT SD	2.0 ± 10.8	2.3	0.87	0.394	0.19
	Variability	1.64 ± 14.9	3.17	0.52	0.661	0.11
	HRT ISI Change	0.0 ± 12.0	2.56	0.0	1.0	0.0
COGO						
	Omissions	4.46 ± 10.6	2.25	1.2	0.61	0.42
	Commissions	−1.45 ± 7.3	1.55	−0.93	0.36	−0.20
	Perseverations	−0.09 ± 16.1	3.44	−0.26	0.979	−0.006
	HRT	1.18 ± 8.15	1.74	1.05	0.307	0.22
	HRT SD	1.18 ± 11.04	2.4	0.50	0.621	0.11
	Variability	−2.05 ± 14.2	3.03	−0.67	0.507	−0.14
	HRT ISI Change	1.68 ± 13.2	2.82	0.6	0.557	0.13
Citicoline						
	Omissions	4.71 ± 14.03	3.1	1.54	0.139	0.34
	Commissions	0.33 ± 8.5	1.85	0.18	0.859	0.04
	Perseverations	4.95 ± 14.8	3.32	1.53	0.142	0.33
	HRT	0.24 ± 6.77	1.5	0.16	0.874	0.35
	HRT SD	2.95 ± 11.4	2.49	1.18	0.25	0.26
	Variability	2.81 ± 9.7	2.11	1.33	0.198	0.29
	HRT ISI Change	3.3 ± 14.2	3.1	1.1	0.301	0.23

HRT: Hit Reaction Time, ISI: Inter-Stimulus Interval, SD: Standard Deviation. Bold values indicate statistical significance at 0.05 level.

**Table 3:** Differences between groups based on continuous performance test scores.

Variable	Sum of Squares	df	Mean Square	F	*p*	Partial η^2^
Omission	164.31	3	54.77	0.31	0.816	0.01
Commission	122.48	3	40.83	0.48	0.694	0.02
Perseveration	830.68	3	276.89	1.78	0.158	0.06
HRT	68.12	3	22.71	0.21	0.891	0.01
HRT SD	463.29	3	154.43	0.86	0.464	0.03
Variability	694.28	3	231.43	1.55	0.209	0.05
HRT Block Change	444.70	3	148.23	1.25	0.297	0.04
HRT ISI Change	353.73	3	117.91	0.88	0.456	0.03

η^2^: Eta Square; df: Degree of Freedom. Post hoc tests were not conducted since no significant differences were detected between the groups. HRT: Hit Reaction Time, ISI: Inter-Stimulus Interval, SD: Standard Deviation.

**Table 4:** Repeated-measures ANOVA for intervention groups.

Variable	Sum of Squares	df	Mean Square	F	*p*	Partial η^2^
SNAP-IV						
Attention Subscale	5.60	3	1.87	0.2	0.926	0.01
Hyperactivity Subscale	15.32	3	5.11	0.4	0.788	0.02
SCT-Scale	59.86	3	19.95	0.7	0.574	0.02
RCADS	888.78	3	296.26	1.8	0.145	0.06
SDQ						
Emotional	11.23	3	3.74	1.4	0.244	0.05
Conduct	2.84	3	0.95	0.5	0.659	0.02
Hyperactivity	8.37	3	2.79	1.2	0.327	0.04
Peer Problems	2.13	3	0.71	0.4	0.774	0.01
Prosocial Behavior	1.85	3	0.61	0.3	0.790	0.01
Total Difficulty	7.93	3	2.64	0.2	0.928	0.01
BRIEF						
Inhibition	119.81	3	39.94	1.9	0.137	0.06
Shifting	23.00	3	7.67	0.5	0.671	0.02
Emotional	31.82	3	10.61	0.9	0.452	0.03
Initiation	29.96	3	9.99	1.9	0.131	0.07
Working Memory	26.81	3	8.94	0.8	0.476	0.03
Planning	69.49	3	23.16	1.2	0.310	0.04
Organization of Materials	14.46	3	4.82	0.7	0.579	0.02
Monitoring	37.97	3	12.66	1.7	0.184	0.06
Behavioral Regulation Index	331.03	3	110.34	1.0	0.376	0.04
Metacognition Index	646.92	3	215.64	1.3	0.294	0.04
Global Executive Composite	1626.74	3	542.25	1.1	0.350	0.04

η^2^: Eta Square; df: Degree of Freedom. SCT: sluggish cognitive tempo; RCADS: The Revised Child Anxiety and Depression Scale; SDQ: Strenghts and Difficulties Questionnaire; BRIEF: Behavior Rating Inventory of Executive Function; SNAP-IV: The Swanson, Nolan, and Pelham Rating Scale Different superscripts show post hoc differences.

## Data Availability

The data presented in this study are available on reasonable request from the corresponding author. The data are not publicly available due to privacy and ethical restrictions, but can be shared upon request with the permission of the authors.

## Supplementary Materials

The following supporting information can be downloaded at: https://www.mdpi.com/article/10.3390/medicina62030448/s1, Table S1: Pre–post comparisons of emotional, behavioral, and executive-function measures across the four treatment groups.
