# Supplementary material for: Comparative Effects of BCI-Based Attention Training, Methylphenidate, and Citicoline on Attention and Executive Function in School-Age Children: A Quasi-Experimental Study
Source: Medicina (Kaunas). 2026 Feb 27;62(3):448. doi: 10.3390/medicina62030448 (PMC13027574; doi:10.3390/medicina62030448)
Supplement: Supplementary file 1 [file medicina-62-00448-s001.zip › medicina-4045892-supplementary.pdf]

COGO+MPH					
SNAP-IV					
	Attention	14.1 ± 5.2	14.0 ± 6.8	0.0	0.969
	Hyperactivity	12.2 ± 5.1	11.6 ± 7.2	0.6	0.524
SCT-Scale		22.2 ± 7.5	20.8 ± 7.8	1.0	0.342
RCADS		32.2 ± 16.3	33.4 ± 18.8	-0.3	0.792
SDQ					
	Emotional	4.2 ± 2.6	3.9 ± 2.3	0.6	0.551
	Conduct	2.7 ± 1.5	2.2 ± 1.5	1.2	0.255
	Hyperactivity	6.6 ± 2.1	5.5 ± 2.3	2.3	**0.030**
	Peer Problems	2.7 ± 2.1	3.0 ± 2.3	-0.7	0.480
	Prosocial Behavior	7.4 ± 1.6	7.0 ± 2.3	0.9	0.378
	Total Difficulty	16.2 ± 4.9	14.6 ± 6.1	1.2	0.229
BRIEF					
	Inhibition	31.0 ± 6.7	28.0 ± 8.1	2.3	**0.032**
	Shifting	23.9 ± 5.0	22.2 ± 4.4	1.5	0.151
	Emotional	20.7 ± 3.8	19.6 ± 4.8	1.0	0.312
	Initiation	17.8 ± 3.2	16.9 ± 4.0	1.3	0.222
	Working Memory	25.1 ± 4.8	23.4 ± 4.6	1.6	0.131
	Planning	34.8 ± 6.5	31.5 ± 7.3	2.7	**0.014**
	Organisation of Materials	18.0 ± 3.8	16.7 ± 4.8	1.5	0.143
	Monitoring	19.1 ± 3.4	17.4 ± 3.7	2.6	**0.021**
	Behavioral Regulation Index	75.6 ± 13.0	70.0 ± 15.7	1.9	0.071
	Metacognition Index	114.7 ± 18.3	106.0 ± 22.0	2.3	**0.031**
	Global Executive Composite	190.3 ± 28.3	175.8 ± 35.7	2.2	**0.036**
Cogo+Citicoline					
SNAP-IV					
	Attention	15.5 ± 6.2	15.0 ± 4.2	0.5	0.609
	Hyperactivity	11.9 ± 5.7	11.7 ± 5.1	0.2	0.878
SCT-Scale		21.6 ± 6.9	22.8 ± 6.9	-0.7	0.496
RCADS		34.5 ± 22.1	24.5 ± 15.6	2.6	**0.016**
SDQ					
	Emotional	4.0 ± 3.2	2.9 ± 2.2	2.4	**0.026**
	Conduct	2.5 ± 1.9	2.5 ± 1.5	-0.1	0.912
	Hyperactivity	6.1 ± 2.1	6.0 ± 1.9	0.3	0.768
	Peer Problems	3.8 ± 2.3	3.7 ± 2.0	0.2	0.822
	Prosocial Behavior	8.4 ± 1.3	7.9 ± 2.0	1.4	0.185
	Total Difficulty	16.4 ± 7.0	15.1 ± 4.8	1.2	0.249
BRIEF					
	Inhibition	28.3 ± 6.3	28.6 ± 5.1	-0.3	0.801
	Shifting	22.3 ± 4.8	21.6 ± 3.1	0.5	0.595
	Emotional	21.0 ± 5.1	19.0 ± 4.1	2.8	**0.012**
	Initiation	16.6 ± 3.9	17.6 ± 3.6	-1.5	0.146
	Working Memory	24.1 ± 5.1	24.0 ±3.9	0.1	0.922
	Planning	33.8 ± 7.4	33.4 ± 6.8	0.3	0.742
	Organisation of Materials	16.9 ± 4.5	17.2 ± 4.9	-0.3	0.731

	Monitoring	18.1 ± 3.5	18.8 ± 2.8	-0.9	0.371
	Behavioral Regulation Index	71.5 ± 14.2	69.2 ± 9.6	0.9	0.384
	Metacognition Index	109.5 ± 20.8	111.0 ± 18.7	-0.4	0.676
	Global Executive Composite	181.0 ± 31.9	180.3 ± 23.5	0.1	0.898
COGO					
	SNAP-IV				
	Attention	14.7 ± 4.7	14.5 ± 4.3	0.2	0.826
	Hyperactivity	13.0 ± 5.4	13.0 ±6.7	0.0	0.966
	SCT-Scale	22.9 ± 7.2	21.1 ± 8.7	0.9	0.394
	RCADS	35.7 ± 16.9	32.8 ± 17.7	0.9	0.393
	SDQ				
	Emotional	4.7 ± 2.4	3.8 ± 2.7	2.0	0.054
	Conduct	3.7 ± 1.9	3.2 ± 2.3	1.1	0.285
	Hyperactivity	6.2 ± 2.3	6.3 ± 2.0	-0.1	0.919
	Peer Problems	3.2 ± 2.2	3.7 ± 2.0	-1.1	0.264
	Prosocial Behavior	7.0 ± 1.9	7.0 ± 1.8	0.0	1.000
	Total Difficulty	17.8 ± 5.8	17.0 ± 6.4	0.6	0.531
	BRIEF				
	Inhibition	31.0 ± 6.6	30.0 ± 7.8	0.8	0.444
	Shifting	23.2 ± 4.1	23.2 ± 4.8	0.0	0.966
	Emotional	22.6 ± 5.4	22.1 ± 5.7	0.4	0.678
	Initiation	17.4 ± 2.8	18.0 ± 2.8	-0.7	0.511
	Working Memory	24.6 ± 4.6	24.2 ± 3.9	0.5	0.635
	Planning	33.7 ± 6.7	33.4 ± 6.0	0.2	0.861
	Organisation of Materials	18.0 ± 3.7	17.6 ± 4.2	0.7	0.476
	Monitoring	19.2 ± 2.6	18.5 ± 3.1	0.7	0.473
	Behavioral Regulation Index	76.8 ± 13.7	75.3 ± 15.6	0.5	0.611
	Metacognition Index	112.9 ± 16.3	111.7 ± 16.6	0.3	0.771
	Global Executive Composite	189.7 ± 24.5	187.0 ± 27.5	0.4	0.682
Citicoline					
	SNAP-IV				
	Attention	14.0 ± 5.1	13.0 ± 5.4	1.0	0.320
	Hyperactivity	11.6 ± 6.5	12.6 ± 5.5	-0.7	0.478
	SCT-Scale	18.8 ± 5.5	18.7 ± 5.5	0.1	0.915
	RCADS	26.0 ± 11.4	26.9 ± 18.0	-0.2	0.810
	SDQ				
	Emotional	3.3 ± 2.1	3.5 ± 2.8	-0.3	0.779
	Conduct	2.9 ± 2.1	2.9 ± 2.1	-0.1	0.906
	Hyperactivity	6.0 ± 2.4	5.3 ± 2.5	1.4	0.176
	Peer Problems	2.8 ± 1.7	2.9 ± 2.0	-0.2	0.839
	Prosocial Behavior	7.7 ± 2.0	7.2 ± 1.9	1.3	0.192
	Total Difficulty	15.0 ± 5.1	14.5 ± 6.2	0.4	0.730
	BRIEF				
	Inhibition	27.8 ± 7.0	29.4 ± 7.6	-0.9	0.360
	Shifting	22.3 ± 4.7	22.5 ± 5.0	-0.1	0.888
	Emotional	20.0 ± 4.2	20.4 ± 6.1	-0.3	0.804
	Initiation	17.3 ± 3.5	16.4 ± 3.8	1.5	0.151
	Working Memory	23.1 ± 4.3	23.6 ± 5.2	-0.5	0.658
	Planning	32.4 ± 5.9	32.0 ± 7.4	0.3	0.798

Organisation of Materials	17.0 ± 4.9	16.8 ± 4.8	0.2	0.836
Monitoring	17.5 ± 3.2	17.9 ± 3.6	-0.4	0.680
Behavioral Regulation Index	70.2 ± 12.9	72.2 ± 16.6	-0.6	0.587
Metacognition Index	107.2 ± 18.3	106.7 ± 22.3	0.1	0.899
Global Executive Composite	177.4 ± 27.6	178.9 ± 37.5	-0.2	0.845

Table S1. Pre–post comparisons of emotional, behavioral, and executive-function measures across the four treatment groups
